# Behavioral, cognitive, and adaptive development in infants with autism spectrum disorder in the first 2 years of life

**DOI:** 10.1186/s11689-015-9117-6

**Published:** 2015-07-16

**Authors:** Annette Estes, Lonnie Zwaigenbaum, Hongbin Gu, Tanya St. John, Sarah Paterson, Jed T. Elison, Heather Hazlett, Kelly Botteron, Stephen R. Dager, Robert T. Schultz, Penelope Kostopoulos, Alan Evans, Geraldine Dawson, Jordana Eliason, Shanna Alvarez, Joseph Piven, IBIS network

**Affiliations:** Department of Speech and Hearing Sciences, University of Washington, Seattle, WA USA; Department of Psychology, University of Washington, Seattle, WA USA; Department of Pediatrics, University of Alberta, Edmonton, AB Canada; Department of Biostatistics, University of North Carolina, Chapel Hill, NC USA; Carolina Institute for Developmental Disabilities, Chapel Hill, NC USA; The Center for Autism Research, Department of Pediatrics, Children’s Hospital of Pennsylvania, University of Pennsylvania, Philadelphia, PA USA; Institute of Child Development, University of Minnesota, Minneapolis, MN USA; Department of Radiology, University of Washington, Seattle, WA USA; Department of Psychiatry, Duke University, Durham, NC USA; Department of Psychiatry, Washington University, St. Louis, MO USA; Montreal Neurological Institute, McGill University, Montreal, QB Canada; The Autism Speaks Foundation, New York, NY USA

## Abstract

**Background:**

To delineate the early progression of autism spectrum disorder (ASD) symptoms, this study investigated developmental characteristics of infants at high familial risk for ASD (HR), and infants at low risk (LR).

**Methods:**

Participants included 210 HR and 98 LR infants across 4 sites with comparable behavioral data at age 6, 12, and 24 months assessed in the domains of cognitive development (Mullen Scales of Early Learning), adaptive skills (Vineland Adaptive Behavioral Scales), and early behavioral features of ASD (Autism Observation Scale for Infants). Participants evaluated according to the DSM-IV-TR criteria at 24 months and categorized as ASD-positive or ASD-negative were further stratified by empirically derived cutoff scores using the Autism Diagnostic Observation Schedule yielding four groups: HR-ASD-High, HR-ASD-Moderate (HR-ASD-Mod), HR-ASD-Negative (HR-Neg), and LR-ASD-Negative (LR-Neg).

**Results:**

The four groups demonstrated different developmental trajectories that became increasingly distinct from 6 to 24 months across all domains. At 6 months, the HR-ASD-High group demonstrated less advanced Gross Motor and Visual Reception skills compared with the LR-Neg group. By 12 months, the HR-ASD-High group demonstrated increased behavioral features of ASD and decreased cognitive and adaptive functioning compared to the HR-Neg and LR-Neg groups. By 24 months, both the HR-ASD-High and HR-ASD-Moderate groups demonstrated differences from the LR- and HR-Neg groups in all domains.

**Conclusions:**

These findings reveal atypical sensorimotor development at 6 months of age which is associated with ASD at 24 months in the most severely affected group of infants. Sensorimotor differences precede the unfolding of cognitive and adaptive deficits and behavioral features of autism across the 6- to 24-month interval. The less severely affected group demonstrates later symptom onset, in the second year of life, with initial differences in the social-communication domain.

**Electronic supplementary material:**

The online version of this article (doi:10.1186/s11689-015-9117-6) contains supplementary material, which is available to authorized users.

## Background

The timing and pattern of symptoms associated with emergence of autism spectrum disorder (ASD) may hold clues for better understanding of the underlying pathogenesis of ASD and lead to earlier identification of affected individuals. Current research indicates that ASD recurrence risk for siblings of children with ASD is as high as 18.7 % [38], with population-based estimates around 10 % [44]. Prospective assessment of high-risk infants can characterize early development, prior to diagnosis, in children who will subsequently develop ASD. This approach provides a unique, real-time window into development in high-risk infants who demonstrate a wide range of outcomes [29].

Previous longitudinal studies of high-risk infant siblings [23, 34, 52] have suggested that ASD onset may be more complex and less homogeneous than previously understood [1, 33, 35], both with respect to timing and the consolidation of symptoms into a clear diagnostic profile [51]. It appears that for most high-risk (HR) infants, the hallmark features of ASD, social-communication deficits, and repetitive behavior are not evident until 12 months of age or later [3, 10, 12, 14, 36, 47, 49]. Yet, there is growing evidence that other developmental differences associated with ASD emerge earlier, in the first year of life. Reduced motor control, as indexed by persistent head lag at 6 months of age, may be characteristic of high-risk infants later diagnosed with ASD [13]. A recent general population survey suggests that parent-reported delays in fine motor and social communication at 6 months may be predictive of a later diagnosis of ASD [2]. Reduced orientation to the eyes and faces in the context of presentation of complex social scenes has also been reported in the first year [6, 19]. However, there is not yet evidence of observable, overt social-communication deficits during the first year of life. Furthermore, whether early atypicalities in the motor and visual attention domains represent non-specific phenomena associated with general risk for developmental difficulties, are specific to vulnerability for ASD, or are prodromal phenomena associated with early manifestations of the disorder itself is unclear. Elucidating the timing and progression of early developmental differences in infants who go on to develop ASD could lead to earlier identification and provide insights into ways of altering the early course of ASD, ultimately identifying novel targets for intervention and opportunities to ameliorate later symptoms.

The present study, part of a multisite Infant Brain Imaging Study (IBIS) Network investigating ASD, evaluated developmental characteristics and behavioral features in infants at high risk for ASD (HR) and a comparison group at low risk for ASD (LR) with typically developing older siblings. In contrast to previous prospective studies, we examined the relationship between early behavior and later outcomes not only based on the presence or absence of an ASD diagnosis but also with regard to symptom severity. HR infants meeting the DSM-IV-TR criteria for ASD (HR ASD-positive) were stratified according to well-established, empirically derived categories on the Autism Diagnostic Observation Schedule (ADOS; [13]). HR ASD-positive infants with higher ADOS scores (i.e., above the ADOS autism cutoff) and lower ADOS scores (i.e., above the ADOS ASD cutoff) were compared to HR and LR infants who did not meet either the DSM-IV-TR criteria or ADOS criteria for ASD. Our objectives were to compare these groups with respect to: (1) longitudinal trajectories of cognitive development and adaptive functioning from 6 to 24 months and cross-sectional differences at 6, 12, and 24 months and (2) behavioral features at 6 and 12 months.

## Methods

### Participants

HR (*n* = 210) and LR (*n* = 98) IBIS participants were included in this study. All participants were screened and excluded based on the following: (1) genetic conditions or syndromes, (2) medical/neurological conditions affecting growth, development, or cognition (e.g., seizure disorder) or significant sensory impairments (e.g., vision or hearing loss), (3) birth weight <2000 g and/or gestational age <36 weeks or significant perinatal adversity and/or exposure in utero to neurotoxins, (4) contraindication for MRI, (5) predominant home language other than English, (6) adopted children or half siblings, (7) first-degree relative with psychosis, schizophrenia, or bipolar disorder (Family Interview for Genetic Studies (FIGS; [28]), and (8) twins. HR infants had a sibling who met the ASD criteria on the Social Communication Questionnaire (SQC; [42]) and Autism Diagnostic Interview (ADI-R; 27]), confirmed by medical records. LR infants had typically developing older siblings who did not meet the autism screening criteria (on the SCQ and FIGS) and no first-degree relative with ASD or intellectual disability. Any questions regarding the inclusion criteria were referred to an expert committee, and a detailed log of specific decisions was kept on a central website accessible to all clinical sites.

### Procedures

Following screening for eligibility, participants were assessed at age 6 months (“6 month visit”), 12 months (“12 month visit”), and 24 months (“24 month visit”). Families were provided a written evaluation and information about intervention services if needed. Written informed consent approved by each site’s Human Subjects Review Board was obtained for all families.

Behavioral features and cognitive development were measured by a licensed clinical psychologist or doctoral student in clinical psychology or school psychologist or masters-level psychometrist under supervision of a licensed clinical psychologist or psychiatrist. Adaptive functioning was evaluated by parent interview. Assessors and parents were necessarily aware of the risk group status of participants, but notably, as with all prospective studies that use future symptom levels to define outcome groups, no one was aware of the ultimate group membership of the participants. All participants were assessed using the Mullen Scales of Early Learning (MSEL; [31]) and the ADOS by examiners meeting research reliability standards. Parent report on the ADI was obtained by research-reliable examiners. A priori examiner training and administration and scoring reliability procedures were implemented to ensure comparability of data across sites. Each participant was assigned a clinical best estimate diagnosis according to the DSM-IV-TR criteria (DSM 5 was not available during the time frame of the study) to determine whether the child met the criteria for Autistic Disorder or Pervasive Developmental Disorder-Not Otherwise Specified by two clinicians: the examiner who conducted the assessment and a senior clinical psychologist or psychiatrist naive to the risk group and previous evaluation results who reviewed the testing results and video for each case to provide an independent confirmation of the DSM diagnosis.

### Measures

#### Cognitive development

The MSEL [31], a standardized, normed, developmental assessment for children’s birth through 68 months, provides an overall index of cognitive ability and delay. The Early Learning Composite (ELC), Receptive Language, Expressive Language, Visual Reception, Fine Motor, and Gross Motor subscales were assessed at each visit.

#### Adaptive functioning

The Vineland Adaptive Behavior Scales-II (VABS-II; [46]) provides a standardized, normed assessment of Communication, Daily Living Skills, and Social and Motor skills. The VABS-II is a semi-structured parent interview completed at each visit to assess children’s behavior in everyday settings. An Adaptive Behavior Composite (ABC) provides an overall index of adaptive function.

#### Early behavioral features

The Autism Observation Scale for Infants (AOSI; [4]) was administered at 6 and 12 month visits. The AOSI examines 16 ASD features: visual tracking and attentional disengagement, coordinated eye gaze and action, imitation, affective responses, early social-communicative behaviors, behavioral reactivity, and sensorimotor development. The current study randomly sampled 54 AOSIs then coded by examiners naive to the participant risk status to assess rater bias. Scores from 6 month (9 LR/12 HR) and 12 month visits (17 LR/16 HR) were not significantly different for naive versus non-naive coders (*p* = 0.39) risk status (*p* = 0.50), visit (*p* = 0.66), or site (*p* = 0.25).

#### Autism symptoms

The ADOS [26] is a semi-structured play assessment of communication, social interaction, play skills, and restricted interests/repetitive behavior. Module 1 was administered to all subjects at 24 months. Empirically derived, conventional scoring algorithms were utilized [15]. Algorithm scores are based on the severity and number of ASD symptoms demonstrated during the ADOS assessment and yield three classifications, Autism, ASD, and Non-Spectrum, corresponding to the HR-ASD-High, HR-ASD-Mod, HR- and LR-Neg groups, respectively.

The ADI-R [43] is a semi-structured interview assessing symptoms of ASD administered at 24 months to all parents of HR infants and all LR infants with clinical concerns. This information was used in the process of assigning a clinical best estimate diagnosis [20].

### Statistical analysis

Data were available from 222 HR and 107 LR participants who enrolled in the study at 6 months and completed both the ADOS and DSM-IV-TR evaluation at 24 months. Outcome group classifications were based on 24 month DSM-IV-TR and ADOS assessments. Children were categorized as high, if they met the DSM-IV-TR criteria for Autistic Disorder or Pervasive Developmental Disorder-Not Otherwise Specified (PDD-NOS) (DSM+) and criteria for Autism on the ADOS [13], moderate (Mod) if they met the criteria for DSM+ and ASD on the ADOS [13], and negative (Neg) if they did not meet the DSM-IV-TR criteria for either Autistic Disorder or PDD-NOS (DSM-Negative) and scored in the Non-Spectrum range on the ADOS.

Four analysis groups were derived based on the classifications above combined with risk status, HR-ASD-High (*n* = 31), HR-ASD-Mod (*n* = 18), HR-Neg (*n* = 161), and LR-Neg (*n* = 98). Two LR groups (LR-ASD-High, *n* = 2; LR-ASD-Mod, *n* = 2) were too small to be analyzed as a separate group. Seventeen children with discordant DSM and ADOS statuses were excluded from the analysis (DSM-/ADOS Autism, *n* = 2 HR, 1 LR; DSM-/ADOS Spectrum, *n* = 7 HR, 4 LR; DSM+/ADOS Non-Spectrum, *n* = 3 HR) as the groups were too small to be analyzed separately, consistent with recent work in this area [4].

Longitudinal response profiles over multiple visits were analyzed using mixed models with repeated measures (MMRM) for each outcome measure, cognitive development (MSEL), adaptive functioning (VABS-II), and AOSI as described in Tables 2, 3, and 4. For each model, the fixed effects of the model included visit, group, and group X visit interaction. The R matrix assumed the structure of diagonal blocks as defined by subject ID and unstructured variance-covariance structure within each block.

Three covariates were included in each MMRM model. Mother’s education was included as a covariate due to its known effect on child development and significant difference between the HR and LR groups. The difference between the child’s actual age at the visit and the scheduled visit age (6, 12, or 24 months) was included as a covariate to account for individual age variation at each visit (see Table 1). Site was entered as a covariate to control for potential population differences at the four data collection sites.

**Table 1:** Sample characteristics at study entry by group

	HR-ASD-High		HR-ASD-Mod		HR-Neg		LR-Neg		*p* ^a^
	(*n* = 31)		(*n* = 18)		(*n* = 161)		(*n* = 98)		(4-Group)
	*n*	%	*n*	%	*n*	%	*n*	%	
Gender									<0.01
Male	26	83.9	15	83.3	88	54.7	55	56.1	
Female	5	16.1	3	16.7	73	45.3	43	43.9	
Ethnicity									0.97
White	27	87.1	15	83.3	140	87.0	84	85.7	
Non-white	4	12.9	3	16.7	21	13.0	14	14.3	
Family income									0.83
Not answered	0	0.0	1	5.6	6	3.7	4	4.1	
<$75,000/year	12	38.7	8	44.4	65	40.4	41	41.8	
> = $75,000/year	19	61.3	9	50.0	90	55.9	53	54.1	
Maternal education									0.02
No college	12	38.7	8	44.4	48	29.8	19	19.4	
College degree	10	32.3	5	27.8	75	46.6	37	37.8	
Graduate degree	9	29.0	5	27.8	38	23.6	42	42.9	
	Mean	Std	Mean	Std	Mean	Std	Mean	Std	
Child gestational age (week)	38.6	1.42	39.1	1.17	39.0	1.24	39.2	1.42	0.41
Age at visits (month)									
6 month visit	6.8	0.82	6.6	0.64	6.8	0.76	6.7	0.71	0.67
12 month visit	13.1	0.88	12.8	0.68	12.9	0.75	12.9	0.76	0.59
24 month visit	24.5	0.80	25.1	2.19	25.1	3.08	25.5	3.1	0.30
ADOS total score at 24 months	17.4	3.53	11.0	2.00	2.6	2.06	2.33	2.0	<0.0001

^a^Fisher’s exact test for categorical variables (gender, family income, maternal education) and ANOVA for continuous variables (gestational age and age at visits). All tests are two-sided at a significance level of .05

A MMRM model was fit with visit and group, all interaction terms, and the three covariates described above for each outcome variable separately. Our primary hypothesis was for diverging longitudinal growth trajectories among the four groups assessed by the interaction term of group X visit in the model. The longitudinal MMRM modeling results are described in the Results section. Estimated least square group means (LSM) and standard error of the mean (SE) at each visit (6, 12, and 24 months) are listed in Tables 2, 3, and 4. The LSM and SE adjust for any effect of mother’s education, age at visit, and site. Overall group differences were tested first and followed by pair-wise comparisons between each pair. LSM and SE were presented in the tables instead of mean and standard deviation to adjust for the group differences in mother’s education, age at visit, and site differences.

**Table 2:** Cognitive development least square means group comparisons at 6, 12, and 24 months

Cognitive development	HR-ASD-High (a)		HR-ASD-Mod (b)		HR-Neg (c)		LR-Neg (d)		Overall Group Comparison^a^				Post hoc comparisons^b^
	(*n* = 31)		(*n* = 18)		(*n* = 161)		(*n* = 98)						
	LSM	SE	LSM	SE	LSM	SE	LSM	SE	df_1_	df_2_	*F*	*p*	
6 Month visit													
MSEL ELC	93.9	1.9	98.3	2.4	98.4	0.8	100.5	1.1	3	299	3.2	0.02	
Expressive Language	42.9	1.3	46.6	1.6	44.5	0.5	46.5	0.7	3	299	3.0	0.03	
Receptive Language	49.6	1.6	47.9	2.1	49.9	0.7	50.0	0.9	3	299	0.3	0.83	
Fine Motor	47.8	1.7	51.2	2.1	50.4	0.7	50.2	1.0	3	299	0.7	0.54	
Gross Motor	43.7	1.5	46.3	2.0	49.0	0.7	50.8	0.9	3	299	5.8	0.0007	a < d
Visual Reception	47.3	1.6	50.5	2.0	51.4	2.0	53.9	0.9	3	299	4.8	0.0028	a < d
12 Month visit													
MSEL ELC	89.2	2.3	93.8	3.1	101.2	1.0	105.5	1.3	3	299	14.5	<0.0001	a < (c, d)
Expressive Language	39.5	2.0	37.7	2.8	47.2	0.9	49.6	1.1	3	299	10.0	<0.0001	(a, b) < d
Receptive Language	36.1	1.5	39.4	2.0	43.5	0.6	45.6	0.8	3	299	11.2	<0.0001	a < (c, d)
Fine Motor	53.9	1.7	57.9	2.3	57.0	0.7	59.6	0.7	3	299	3.4	0.02	
Gross Motor	42.7	2.2	45.6	3.0	49.7	1.0	50.4	1.2	3	299	3.7	0.01	
Visual Reception	47.6	1.7	51.4	2.3	54.3	0.7	55.6	0.9	3	299	6.2	0.0004	a < d
24 Month visit													
MSEL ELC	75.8	3.0	84.1	3.9	103.1	1.3	109.4	1.7	3	299	38.0	<0.0001	(a, b) < (c, d)
Expressive Language	36.1	2.1	39.1	2.7	49.0	0.9	51.7	1.2	3	299	18.3	<0.0001	a < (c, d), b < d
Receptive Language	31.1	2.0	38.8	2.6	51.9	0.9	56.0	1.1	3	299	44.9	<0.0001	(a, b) < (c, d)
Fine Motor	39.9	1.7	42.9	2.3	49.9	0.8	54.6	1.0	3	299	21.6	<0.0001	a < (c, d), (b, c) < d
Gross Motor	38.4	1.6	43.9	2.1	50.2	0.7	52.0	0.9	3	299	19.8	<0.0001	a < (c, d)
Visual Reception	39.9	2.0	45.0	2.6	54.9	0.9	55.9	1.1	3	299	20.4	<0.0001	a < (c, d), b < d

*MSEL* Mullen Scales of Early Learning, *ELC* Early Learning Composite

^a^The Overall Group Comparison serves as an omnibus test comparing the means between the 4 groups to determine whether group means differ based on a mixed model with repeated measures. Model covariates included the difference between the child’s actual age at the visit from the scheduled visit age (6, 12, or 24 months). Other covariates included site and mother’s education. Two-sided at significance level of .05

^b^Post hoc comparisons with step-up Bonferroni correction. Two-sided at significance level of .05

**Table 3:** Adaptive functioning group least square means comparisons at 6, 12, and 24 months

Adaptive functioning	HR-ASD-High (a)		HR-ASD-Mod (b)		HR-Neg (c)		LR-Neg (d)		Overall Group Comparison^a^				Post hoc comparisons^b^
	(*n* = 31)		(*n* = 18)		(*n* = 161)		(*n* = 98)						
	LSM	SE	LSM	SE	LSM	SE	LSM	SE	df_1_	df_2_	*F*	*p*	
6 Month visit													
VABS-II ABC	87.8	1.9	91.9	2.5	95.1	0.9	97.4	1.1	3	299	6.6	0.0003	a < d
Social	92.5	2.2	97.3	2.8	99.2	0.9	101.5	1.2	3	299	4.4	0.0045	
Communication	92.7	2.6	92.5	3.4	98.1	1.2	98.7	1.5	3	299	2.1	0.0947	
Motor	84.3	2.3	91.5	3.0	91.6	1.0	95.5	1.3	3	299	6.1	0.0005	a < d
Daily Living Skills	89.4	2.3	92.7	3.0	95.5	3.0	96.0	1.3	3	299	2.5	0.0622	
12 Month visit													
VABS-II ABC	87.1	1.8	92.4	2.3	97.5	0.8	101.1	1.0	3	299	17.7	<0.0001	a < (c, d)
Social	89.3	1.8	94.8	2.5	98.8	0.8	100.5	1.0	3	299	10.5	<0.0001	
Communication	86.2	2.1	91.8	2.8	99.0	0.9	102.7	1.7	3	299	17.9	<0.0001	a < (c, d)
Motor	94.1	1.9	99.2	2.5	100.7	0.8	103.3	1.0	3	299	6.1	<0.0001	a < d
Daily Living Skills	87.7	1.9	89.5	2.6	94.2	0.8	98.4	1.0	3	299	10.1	<0.0001	a < d
24 Month visit													
VABS-II ABC	84.5	1.6	91.0	2.0	101.2	0.7	105.0	0.9	3	299	48.2	<0.0001	(a, b) < (c, d)
Social	83.7	1.7	91.5	2.1	100.6	0.7	103.0	1.0	3	299	38.0	<0.0001	(a, b) < (c, d)
Communication	84.2	1.8	90.8	2.2	101.9	0.8	105.2	1.0	3	299	42.9	<0.0001	(a, b) < (c, d)
Motor	92.9	1.7	95.1	2.1	100.3	0.7	102.8	0.9	3	299	10.9	<0.0001	a < (c, d)
Daily Living Skills	87.6	1.7	93.7	2.1	102.4	0.7	106.6	0.9	3	299	36.7	<0.0001	(a, b) < (c, d)

*VABS-II* Vineland Adaptive Behavior Scales, *ABC* Adaptive Behavior Composite

^a^The Overall Group Comparison serves as an omnibus test comparing the means between the 4 groups to determine whether group means differ based on a mixed model with repeated measures. Model covariates included the difference between the child’s actual age at the visit from the scheduled visit age (6, 12, or 24 months). Other covariates included site and mother’s education. Two-sided at significance level of .05

^b^Post hoc comparisons with step-up Bonferroni correction. Two-sided at significance level of .05

**Table 4:** Autism behaviors longitudinal response profile and least square means by group at 6 and 12 months

AOSI total score	HR-ASD-High (a)		HR-ASD-Mod (b)		HR-Neg (c)		LR-Neg (d)		Overall Group Comparison^a^				Post hoc comparisons^b^
	(*n* = 31)		(*n* = 18)		(*n* = 161)		(*n* = 98)						
	LSM	SE	LSM	SE	LSM	SE	LSM	SE	df_1_	df_2_	*F*	*p*	
6 Month visit	10.6	0.7	10.1	0.9	9.7	0.3	8.2	0.4	3	293	4.1	0.0068	
12 Month visit	9.3	0.7	7.9	0.9	5.1	0.3	4.4	0.4	3	293	15.8	<0.0001	a < (c, d)

^a^The Overall Group Comparison serves as an omnibus test comparing the means between the four groups to determine whether group means differ based on a mixed model with repeated measures. Model covariates included the difference between the child’s actual age at the visit from the scheduled visit age (6 and12 months). Other covariates included site and mother’s education. Two-sided at significance level of .05

^b^Post hoc comparisons with step-up Bonferroni correction. Two-sided at significance level of .05

All tests and corresponding *p* values were two-sided. Post hoc group comparison results for all outcome variables were adjusted for multiple comparisons using step-up Bonferroni adjustments, also called Hochberg-adjusted *p* values [17].

The analysis used SAS 9.3 statistics software (SAS Institute Inc., Cary, NC, USA).

## Results

Demographic information on the sample appears in Table 1. There were more boys in the HR-ASD-High and HR-ASD-Mod groups as compared with the HR-Neg and LR-Neg groups, consistent with the sex ratio of children with ASD in the general population.

### Cognitive development

At 6 months, the HR-ASD-High group had significantly lower MSEL Gross Motor and Visual Reception scores compared with the LR-Neg group (*t*(299) = −3.97, *p* = 0.014; *t*(299) = −3.65, *p* = 0.046). Neither ELC nor other subscales showed significant group differences at 6 months (see Table 2). Notably, the HR-ASD-Mod group did not differ from the HR-Neg and LR-Neg groups in any respect at 6 months.

At 12 months, the HR-ASD-High group demonstrated significantly lower MSEL ELC and Receptive Language scores than the HR-Neg and LR-Neg groups (ECL: *t*(299) = −4.77, *p* < 0.0005 and *t*(299) = −6.15, *p* < 0.0001; Receptive Language: *t*(299) = −4.46, *p* < 0.002 and *t*(299) = −5.47, *p* < 0.0001). The HR-ASD-High group also had significantly lower Visual Reception and Expressive Language scores than LR-Neg (*t*(299) = −4.14, *p* < 0.01, *t*(299) = −4.33, *p* < 0.005). This was the first age point at which the HR-ASD-Mod group differed and was significantly lower than the LR-Neg group on the Expressive Language subscale (*t*(299) = −4.46, *p* < 0.002).

At 24 months, the HR-ASD-High group had lower scores on ELC and all subscales compared with the HR-Neg and LR-Neg groups (*p* < 0.001 for all comparisons). The HR-ASD-Mod group had lower scores on the ELC and Receptive Language scales compared with the HR-Neg and LR-Neg groups and lower Fine Motor, Visual Reception, and Expressive Language scores compared with the LR-Neg group.

**Fig. 1:**
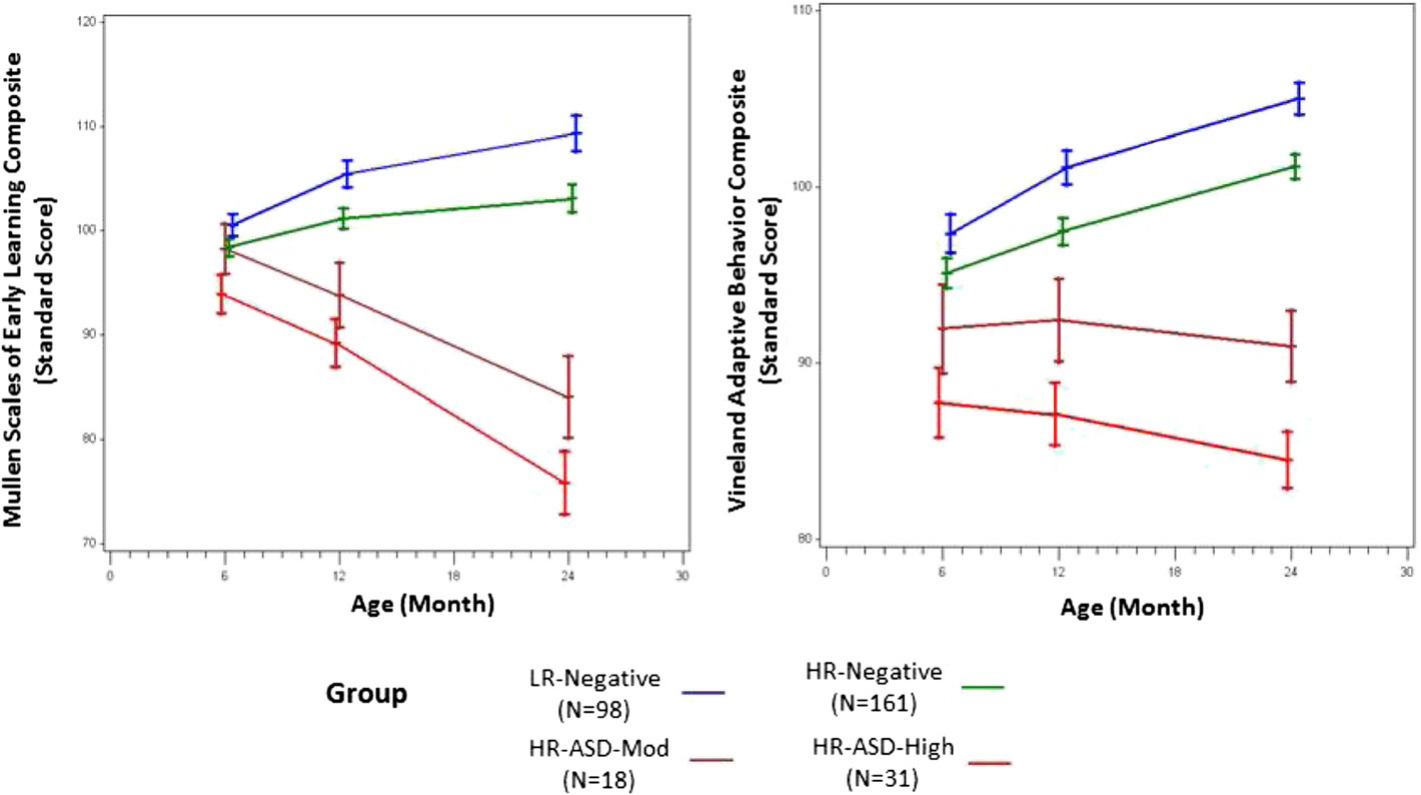
Cognitive and adaptive functioning at 6, 12, and 24 months

Longitudinal trajectories from 6 to 24 months showed significant overall group differences on MSEL ELC and all subscales (*p* < 0.0001; see Fig. 1 and Additional file 1: Table S1). Group differences in the motor domain did not continue to increase over time (Gross Motor *F* = 1.74, *p* = 0.11), but for the ELC and all other subscales, the group differences increased over time, with significant interaction terms (*p* < 0.001). Group means at 6, 12, and 24 months reveal decreasing standard scores on the ELC in the HR-ASD-High and HR-ASD-Mod groups and stable or increasing scores across time in the HR-Neg and LR-Neg groups (Fig. 1).

### Adaptive functioning

At 6 months, post hoc comparisons revealed that the HR-ASD-High group had significantly lower ABC and Motor scores than the LR-Neg group (Table 3; *t*(299) = −4.27, *p* < 0.005 and *t*(299) = −4.21, *p* < 0.005, respectively). No other significant group differences were evident at 6 months, including with the HR-ASD-Mod group.

At 12 months, post hoc comparisons revealed the HR-ASD-High group had decreased ABC and Communication scores compared with HR-Neg (*t*(299) = −5.41, *p* < 0.0001; *t*(299) = −5.62, *p* < 0.0001) and LR-Neg (*t*(299) = −6.95, *p* < 0.0001; *t*(299) = −6.91, *p* < 0.0001). The HR-ASD-High group also had lower Motor and Daily Living Skills scores than LR-Neg (*t*(299) = −4.22, *p* < 0.01; *t*(299) = −4.90, *p* < 0.001). Only the Social subscale did not differ at 12 months.

At 24 months, post hoc comparisons revealed the HR-ASD-High group showed significantly lower scores on the ABC and all subscales compared with the HR-Neg and LR-Neg groups (*p* < 0.0001 in all comparisons). The HR-ASD-Mod group had lower scores than HR-Neg and LR-Neg on the ABC and all subscales except Motor.

VABS-II longitudinal trajectory analysis from 6 to 24 months demonstrated a pattern similar to the MSEL (Fig. 1; Additional file 1: Table S1). The four groups differed significantly from one another on the ABC and all subscales (*p* < 0.0001). These differences increased from 6 to 24 months on the ABC and all subscales (*p* < 0.001) except for Motor. The significant Motor group differences were stable over time (group X visit interaction; *F*(6, 299) = 1.74, *p* = 0.11).

### Behavioral features on AOSI

There was no evidence of group differences at 6 months (Table 4). The HR-ASD-High group showed significantly higher scores on the AOSI than HR-Neg and LR-Neg at 12 months with corrected, post hoc comparisons (*t*(293) = 5.68, *p* < 0.0001, *t*(293) = 6.18, *p* < 0.0001).

Longitudinal trajectories from 6 to 12 months showed that the HR-ASD-High group had overall higher AOSI total scores compared with the HR-Neg and LR-Neg groups (*F*(3,293) = 13.8, *p* < 0.0001). The significant group X visit interaction term suggests that differences increased between 6 and 12 months (*F*(3, 293) = 4.5, *p* < 0.005).

## Conclusions

This study demonstrated robust differences at 6 months on standardized developmental measures in high-risk infants who go on to develop ASD at 24 months. These early decrements in cognitive and adaptive function were specific to the HR-ASD-High group compared to the LR-Neg group in the Gross Motor and Visual Reception domains on the MSEL and Motor domain on the VABS-II. There was no evidence of atypical social-communication or repetitive behavior at 6 months in the HR-ASD-High group, as indexed by the AOSI, but such features were evident at 12 months relative to other HR and LR infants. Notably, most cognitive and adaptive skill differences between the HR-ASD-Mod and non-ASD groups emerged at 24 months (with the exception of lower MSEL Expressive Language which emerged at 12 months).

Longitudinal trajectory findings revealed significant increasing group differentiation over time, from 6 to 24 months, across all domains (cognitive, adaptive, and behavioral features). A hierarchical pattern of symptom severity was observed with regard to mean group differences and developmental trajectories across all developmental domains: cognitive, adaptive, and behavioral features, with the HR-ASD-High group being the most severely affected at each age, followed by the HR-ASD-Mod, HR-Neg, and LR-Neg groups. Taken together, these data illustrate a pattern of unfolding symptoms in children with ASD, starting in the sensorimotor domain at 6 months and moving to the social-communication domain in the second year of life.

This unfolding is also reflected in the unique trajectories of the HR-ASD-Mod group. The HR-ASD-Mod group differed from the HR-ASD-High group demonstrating symptoms that emerged later in development, less severe behavioral features, and more advanced cognitive and adaptive functioning at 24 months. The more severely affected group presented initially with lower sensorimotor abilities relative to the non-ASD groups at 6 months, whereas the less severely affected subgroup demonstrated differences relative to the non-ASD groups at 12 months in the communication domain. Both the HR-ASD-High and the HR-ASD-Mod groups demonstrated increasing severity of cognitive and adaptive functioning deficits in the first 2 years of life. A follow-up with these children is underway to investigate diagnostic trajectories and patterns of symptom expression in the preschool and early school-age years.

There was a remarkable convergence across parent-report and direct clinical assessment identifying atypical motor development as early as 6 months in children with elevated ASD symptoms at age 2, suggesting motor development, which is not a diagnostic feature, may be disrupted very early in development in ASD. Indeed, motor and visual reception differences were detected earlier than reported in previous longitudinal studies of high-risk infants that used standardized developmental measures [21, 34]. Our findings are consistent with reports of subtle motor differences at 6–7 months with respect to head control [13], lower activity level [52], and saccadic reaction times [9] among infants who go on to develop ASD. Early motor deficits could play a role in later motor impairments, atypical control of eye movements, and/or delays in development of gestures such as pointing, associated with the emergence of joint attention, a commonly reported deficit in ASD. A dynamic system perspective suggests that motor and language development are intimately linked during infancy and toddlerhood, as emerging voluntary control over oculomotor, fine motor, and gross motor systems allows for increasingly complex interactions with one’s environment [18]. Notably, motor differences evident at 6 months did not increase over time (i.e., the longitudinal trajectory interaction term was not significant), and at 12 months, outcome groups did not differ. The pattern of group differences was similar when utilizing the VABS Motor scale or the VABS Gross Motor V scale (data available on request.) Future studies should clarify whether this pattern of motor development, in which groups diverge at 6 months and converge at 12 months, reflects qualities of the measurement tools or developmental processes related to the onset of ASD. It is also possible that differences identified at 6 months may be signs of risk for developmental disorders more generally, or comorbid intellectual disability, rather than precursors of ASD specifically. The defining behaviors of ASD (e.g., atypical or delayed verbal communication, lack of pretend play, poor reciprocal social interaction) begin to emerge around 12 months and are increasingly evident over the second year of life. Commonly used measures of social communication may not be designed detect the kinds of aberrant social-communicative behavior that emerge in the first year of life. Advances in measurement, particularly in the social-communication domain, may be needed to capture very early ASD-related behavioral manifestations.

Recent evidence, also from the IBIS network, demonstrates differences in DTI white matter fiber tract trajectories at 6 months of age [50]. These findings utilized the same research design and a subset of the sample reported here to characterize brain differences in infants who met the ADOS criteria for ASD (HR-ASD-High and HR-ASD-Mod combined) at 24 months compared with infants who did not (HR-Neg). The timing of behavioral differences observed in the expanded HR-ASD-High group reported here is consistent with the onset white matter tract differences, both observed at 6 months. Furthermore, the brain and behavioral differences in these reports both appear to unfold in a dynamic manner over the 6- to 24-month period. The precise nature and evolution of these differences requires further examination with more granular assessments delineating trajectories with greater precision and with varying comparison groups to determine whether these differences are specific to ASD or more general developmental risk factors. But, in combination, these data suggest a process of neurobehavioral alteration that begins early in infancy and unfolds over time.

This longitudinal, prospective study was a large-scale replication of previous studies and involved one of the largest number of infants reported to date who present with autism symptoms at 24 months (*n* = 49). This provided the opportunity to better represent the heterogeneity in symptom severity through the use of the full range of ADOS classifications. The prospective approach has the added benefit that all data were obtained and scored blind to the ultimate group status, reducing rater bias that could amplify group differences. This study was also the first to our knowledge to assess rating bias related to examiner awareness of risk status on an observational measure of early behavioral features of ASD, the AOSI. This is a previously unexamined methodological issue in existing studies involving non-blind comparison of high- and low-risk infants.

We also note several important limitations. A recent report on high-risk infants who do not go on to develop ASD, followed through 36 months, suggests that over a quarter of high-risk infants without a diagnosis of ASD will nonetheless demonstrate significant developmental difficulties such as ASD symptoms or lower developmental skills [37]. Thus, a follow-up of high-risk infants into preschool and school-age is clinically indicated and may reveal additional information about developmental trajectories, risk, and protective factors in ASD. There is a lack of consensus in the field about the interpretation of 24-month outcomes as compared with later outcomes in studies of infants at high risk for ASD. There is substantial precedent for early diagnoses of ASD in clinically ascertained samples with a high level of diagnostic stability at 24 months (e.g., [5, 16, 24, 25]). Classifying ASD in high-risk sibling cohorts at 24 months is also commonly reported (e.g., [10, 21, 30, 32, 49, 52]). However, there is concern that infants ascertained based on high familial risk may differ from similar-aged clinically ascertained children with ASD. In particular, careful prospective assessment may detect milder symptoms in high-risk infant siblings than studies using clinically referred samples. Nonetheless, high-risk infant sibling studies have reported high classification stability from 24 to 30/36 months (e.g., [19, 39, 41, 45]). There may be a proportion of high-risk children who fail to meet diagnostic thresholds for ASD earlier but go on to later meet the criteria for ASD [7, 39]. In one recent study, the high-risk children not diagnosed at 24 months but subsequently diagnosed at 36 months displayed subthreshold ASD symptoms at 24 months (e.g., ADOS Social Communication score mean 9.2) but met threshold at 36 months (e.g., ADOS Social Communication score mean 11.6) [39]. Since our outcome groups are based on cases who were diagnosed at 24 months of age, these findings may not generalize to cases in which a diagnosis is established later. This subset of children, if present in our sample, may reduce observed group differences because children with subthreshold ASD may be included in the non-ASD group. It has also been observed that a small subset of high-risk 2-year-olds who are classified as having ASD no longer meet the criteria later in development. A common interpretation is that this change reflects a misclassification at 24 months. However, it is perhaps more plausible that this could reflect the natural progression of ASD in these children or varied early experiences such as participation in high-quality early intervention or enriched social-communicative environments. Although it is well known that an ASD diagnosis had strong predictive validity, especially in comparison with other childhood psychiatric diagnoses, this should not be taken to suggest that an ASD classification, especially in very young children, is immutable [48]. Within the field of psychiatric nosology, there is little evidence for permanent diagnoses in childhood conditions. Longitudinal work has highlighted the transience of most psychopathology and the resilience of a large subgroup of children [40]. In sum, changes in symptom expression over the course of a psychiatric disorder should not be unexpected or invalidate a diagnosis at an earlier age.

A third caution is that group differences in motor skills at 6 months on the MSEL and VABS-II represent mild decrements in test scores, rather than frank developmental delays. As well, it is premature to generalize motor findings from a high-risk sample to ASD surveillance/screening efforts in the general population, as motor delays may indicate risk for a broad range of developmental disorders. Rather, we propose that subtle atypicalities in sensorimotor development at 6 months, followed by more pronounced developmental delays and behavioral signs, may inform risk profiles for ASD. Although we interpret the Visual Reception domain differences at 6 months as likely representing differences in the sensory domain and 12-month differences on the Communication domain (VABS-II) and Expressive Language subscale (MSEL) as relating to language abilities, future studies of sensory and language abilities will be needed to replicate and extend these findings. Future research is needed to evaluate the utility of such risk profiles for individual classification. Critically, investigation of combined bio-behavioral markers, utilizing measures of brain imaging, early attention, and other infant-specific approaches may enhance risk prediction over and above present capabilities. Models to improve early, accurate identification of ASD would allow earlier intervention at a time when neuroplasticity is highest [8, 22]. Efforts to develop interventions for children at this early stage of development, perhaps targeting very early differences in sensorimotor or visual tracking, are needed. For families with a history of ASD, earlier and more accurate understanding of ASD behavioral features could reduce parent stress and improve family adaptive functioning by reducing uncertainty [11] and allowing parents to act early to ensure the best possible outcome for their child.

## Additional file

Additional file 1: Table S1. Cognitive development and adaptive functioning longitudinal response profile comparison from 6 to 24 months.
