# Supplementary material for: Behavioral, cognitive, and adaptive development in infants with autism spectrum disorder in the first 2 years of life
Source: J Neurodev Disord. 2015 Jul 16;7(1):24. doi: 10.1186/s11689-015-9117-6 (PMC4511527; doi:10.1186/s11689-015-9117-6)
Supplement: Additional file 1: Table S1. — Cognitive development and adaptive functioning longitudinal response profile comparison from 6 to 24 months. [file 11689_2015_9117_MOESM1_ESM.doc]

Supplemental Table. Cognitive Development and Adaptive Functioning Longitudinal Response Profile Comparison from 6-24 Months

|  | | | **Overall Group ^(a)^**  **Comparison** | | | |
| --- | --- | --- | --- | --- | --- | --- |
|  |  |  | df_1_ | df_2_ | F | P |
| **Cognitive Development** | | |  |  |  |  |
| Group Effect | | |  |  |  |  |
|  | MSEL ELC ^(b)^ | | 3 | 299 | 37.83 | <0.0001 |
|  | | Expressive Language | 3 | 299 | 21.17 | <0.0001 |
|  | | Receptive Language | 3 | 299 | 35.16 | <0.0001 |
|  | | Fine Motor | 3 | 299 | 12.92 | <0.0001 |
|  | | Gross Motor | 3 | 299 | 16.42 | <0.0001 |
|  | | Visual Reception | 3 | 299 | 22.02 | <0.0001 |
| Group X Visit Interaction | | |  |  |  |  |
|  | MSEL ELC ^(b)^ | | 6 | 299 | 10.05 | <0.0001 |
|  | | Expressive Language | 6 | 299 | 6.96 | <0.0001 |
|  | | Receptive Language | 6 | 299 | 12.10 | <0.0001 |
|  | | Fine Motor | 6 | 299 | 5.73 | <0.0001 |
|  | | Gross Motor | 6 | 299 | 1.74 | 0.11 |
|  | | Visual Reception | 6 | 299 | 4.03 | 0.0007 |
| **Adaptive Functioning** | | |  |  |  |  |
| Group Effect | | |  |  |  |  |
|  | VABS-II ABC ^(c)^ | | 3 | 299 | 37.97 | <0.0001 |
|  | | Social | 3 | 299 | 30.79 | <0.0001 |
|  | | Communication | 3 | 299 | 26.11 | <0.0001 |
|  | | Motor | 3 | 299 | 14.52 | <0.0001 |
|  | | Daily Living Skills | 3 | 299 | 25.47 | <0.0001 |
| Group X Visit Interaction | | |  |  |  |  |
|  | VABS-II ABC ^(c)^ | | 6 | 299 | 4.35 | 0.0003 |
|  | Social | | 6 | 299 | 3.65 | 0.0016 |
|  | Communication | | 6 | 299 | 3.93 | 0.0008 |
|  | Motor | | 6 | 299 | 0.52 | 0.79 |
|  | Daily Living Skills | | 6 | 299 | 4.35 | 0.0003 |

^(a)^ Mixed model with repeated measures. Model covariates included the difference between the child’s actual age at the visit from the scheduled visit age (6 month, 12 month or 24 month). Other covariates included site and mother’s education. Two-sided at significance level of .05.

^(b)^ MSEL ELC = Mullen Scales of Early Learning, Early Learning Composite.

^(c)^ VABS-II ABC = Vineland Adaptive Behavior Scales, Adaptive Behavior Composite.
